# Widening participation to medicine: a student-led workshop for medical school applicants

**DOI:** 10.15694/mep.2018.0000130.1

**Published:** 2018-06-13

**Authors:** Ben Ryan, Amy Kitchen, Amy Chan, Holly Gibson, Enam Haque

**Affiliations:** 1 University of Manchester

**Keywords:** Widening Participation, Widening Access, Social Mobility, Medical School Admissions

## Abstract

This article was migrated. The article was marked as recommended.

Context

Widening participation to medicine aims to remove barriers to medical education for under-represented groups. This study examined the impact of a widening participation workshop for medical school applicants. The intervention was delivered by a team of medical student volunteers operating in the North West of England: Manchester Outreach Medics.

Methods

39 pupils, typically aged 16-17, attended the workshop at Royal Blackburn Hospital, Lancashire. Activities included a variety of lectures and interactive group sessions. Using questionnaires, participants highlighted their understanding in areas relating to the medical school application process on a scale of zero to ten. This was performed before and after the intervention to allow for comparison. The results were evaluated using a one-tailed paired t-test and Cohen’s *d* effect size.

Results

There was a significant improvement ( *p* <0.001, Cohen’s *d* >0.8) in the understanding of all topics assessed. The largest improvements were seen in topics that the participants had little understanding in prior to attending the workshop.

Conclusions

This student-led activity improved participant understanding in areas pertaining to the medical school application process. The study also showed the effectiveness of pre- and post-intervention questionnaires, which could be used by all post-16 medical widening participation activities in the UK.

## Introduction

The medical profession in the United Kingdom is not representative of the population it serves ( HEE, 2014). Individuals from deprived backgrounds are less likely to apply to medical school and have a successful application ( Steven et al., 2016). In the General Medical Council survey of Foundation Year One Trainees in 2014, more than half were found to come from the two least deprived quintiles in the country ( MSC, 2014). Widening participation (WP) initiatives look at removing barriers to higher education for under-represented groups, including those from deprived backgrounds ( OFFA, 2017). This is important because medical students who train in more diverse medical schools may gain a deeper understanding of different sociocultural backgrounds, enhancing their ability to provide healthcare to a multicultural population ( Cleland et al., 2013). WP initiatives may also have positive effects on the recruitment of doctors to under-served areas, as doctors from less affluent backgrounds are more likely to serve highly deprived communities ( Hutt & Gilmour, 2010; Dowell et al., 2015).

WP programmes can be classified based on their design; these may be low or high-intensity, and outreach or inreach. Outreach involves delivering activities outside of the host institution in order to provide information and guidance to young people. Open days and campus-based events are examples of inreach interventions which aim to guide university choices. High-intensity programmes offer longitudinal support, and are often tailored towards individuals; they may be associated with guaranteed interviews or foundation programmes ( Cleland et al., 2013).

Many initiatives target pupils from lower socioeconomic backgrounds by considering either the region in which they live or their family background. For example, participants may be selected if they have no close family members who have attended university. This facilitates the recruitment of the target population and attempts to provide equal opportunities and support on par to those who do not come from disadvantaged backgrounds ( Greenhalgh et al., 2006; Garlick & Brown, 2008; Hamdan & Lea, 2012; Curtis et al., 2014; MSC, 2016).

Many WP initiatives are limited by participant selection or intervention delivery. There may be a limited number of places ( Greenhalgh et al., 2006; Hamdan & Lea, 2012), or a large financial cost associated with running the initiative ( MSC, 2016). There may be an element of bias, where the information provided to pupils relates directly to the host medical school. This may fail to include advice regarding applications to other medical schools ( Ratneswaran et al., 2015).

Significant barriers may exist for those from deprived backgrounds applying to medical school ( Mathers & Parry, 2009; Cleland et al., 2013):

• Financial challenges;
• Insufficient academic attainment;
• Lower aspirations;
• Pressure to conform to cultural norms;
• Less knowledge of a career in medicine;
• Lack of support within school environments.

The Medical Schools Council (MSC) advises that post-16 pupils should be supported in applications to medical school. This may include building confidence and teaching the skills and knowledge required. They also advise medical students to act as ambassadors for leading WP initiatives ( MSC, 2014a). Feedback from previous WP activities has highlighted the value of medical students as mentors: improved confidence and aspirations for young people in pursuit of a medical career ( Greenhalgh et al., 2006; Hamdan & Lea, 2012).

In March 2017, a conference for student-led WP was delivered by the MSC. The engagement of 72 medical students from 30 medical schools demonstrated that medical students are involved, and interested, in leading WP activities ( MSC, 2017).

There is currently a drive to improve evaluation and reporting of WP initiatives ( HEE, 2014; MSC, 2014a). However, a literature review performed for this paper identified only four journal articles on the topic of student-led medical WP ( Day et al., 2005; Kamali et al., 2005; Ratneswaran et al., 2015; Ojha & Patel, 2017). Of these, only one was a research article ( Kamali et al., 2005). Ojha & Patel (2017) demonstrated a model for student-led WP groups, and highlighted their organisation structure. However, the efficacy of their interventions was not shown. There remains the need to identify the effectiveness, if any, of student-led initiatives.

Manchester Outreach Medics (MOMs) is a WP initiative created by medical students at The University of Manchester Medical School. MOMs was set up in September 2015 with the aim of supporting young people from less affluent backgrounds who are considering a career in medicine. This has been achieved through the provision of free, informative outreach workshops by medical student volunteers. Ten workshops catering for over 500 pupils in Greater Manchester and Lancashire have been delivered. This study examined the impact of one such workshop held at Royal Blackburn Hospital in November 2016. The workshop was open to year 12 pupils in East Lancashire. This area was among those identified by the MSC as a “cold spot” due to lack of engagement with medical school WP activities ( MSC, 2016). Lancashire has also been recognised to be a deprived area according to the English Indices of Deprivation 2015 ( DCLG, 2015): Lancashire was ranked the 8 ^th^ most deprived Local Enterprise Partnership in England, out of 39. Of the 20 local authority districts with the highest proportion of deprived neighbourhoods in England, 3 were situated in Lancashire.

### Study Objectives

The study’s primary objective was to identify the impact of a medical student-led WP initiative on participant understanding of topics important for the medical school application process.

## Methods

MOMs was established with the support of East Lancashire Hospitals NHS Trust, The University of Manchester Medical School and Access All Areas (AAA). AAA is a department within The University of Manchester Students’ Union which supports student-led widening participation activities. This intervention was funded by The University of Manchester Students’ Union. All volunteers underwent appropriate training which included the AAA safeguarding policy. All volunteers had enhanced disclosure and barring service clearance.

### Intervention Design

MOMs had previously delivered multiple similar workshops. The workshop format was selected as it allowed a large capacity and a variety of teaching styles. Feedback from previous workshops aided in designing the itinerary. Through discussion amongst MOMs members, aims were established:

• Widen participation to medicine by supporting year 12 pupils from a deprived area.

• Improve participant understanding and insight into important areas for medical school applicants.

The intervention was designed to be stimulating and informative, incorporating a mixture of lectures and interactive sessions. Elements of the University of Manchester Medical School programme, such as problem-based learning (PBL) and communication skills with simulated patients, were included in the workshop. These sessions were accompanied by lectures outlining the medical school application process. The itinerary is available on Appendix Item 1.

The workshop was held in November 2016 at the Royal Blackburn Hospital Learning Centre, and was delivered by 22 volunteers.

### Participant Recruitment

An online public page was created via ‘Eventbrite’, which included relevant information about the intervention and a hyperlink to reserve a free ticket. The target audience was clearly identified as Year 12 pupils (typically aged 16-17) considering a career in medicine. Email invitations were sent to 21 sixth forms in the Lancashire region, in particular those in nearby East Lancashire. Invitations were also advertised on East Lancashire Hospital’s website and social media pages. 39 participants attended the intervention.

### Data Gathering

Non-mandatory anonymous questionnaires were given to participants immediately before and after the intervention. The questionnaires gathered data pertaining to participant understanding and participant school type. These were paired using codes given to participants on arrival. Gathered data was collated to obtain quantitative evaluation of the intervention.

### Participant School Type

The aim was to engage a high proportion of participants from state schools. However, the intervention was open to all sixth forms as it was not expected to reach capacity. Participants documented the school they attended. Schools were grouped as ‘state schools’, ‘selective schools’, or ‘fee-paying schools’. ‘State schools’ do not use admissions test, and are free to attend. ‘Selective schools’ use admissions tests in selecting their pupils, and are free to attend. ‘Fee-paying schools’ are schools at which pupils pay a fee to attend. This data was used to analyse the schools successfully engaged, compared to the schools the intervention was advertised to. All selective schools in this study were grammar schools; none used admissions tests at the stage of sixth form entry, but instead at an earlier age.

### Participant Understanding

Volunteers identified important topics relevant to the medical school application process, consistent with the aims of the intervention. Identified topics can be found on Table 1. Questionnaires were designed to compare the pre- and post-intervention understanding of such topics. This was done using a scoring scale of 0 to 10, with 0 representing ‘no understanding’ and 10 representing ‘great understanding’. The means were calculated for pre- and post- intervention scores for each topic. A one-tailed paired-t test was used to assess for a significant improvement in participant understanding. The null hypothesis stated that there was no significant increase in their levels of understanding. Cohen’s *d* was used to calculate the effect size of any change, as this may demonstrate a better estimate of effect than *p*-values alone ( McGough & Faraone, 2009). Cohen’s *d* was calculated for each topic by dividing the difference between the two means of participant understanding, by the pooled standard deviation. This was performed using the Centre for Evaluation and Monitoring’s “Effect Size Calculator” ( CEM, 2017). Interpretation of Cohen’s *d* is often 0.2 ≤ small effect size, 0.5 ≤ medium effect size, 0.8 ≤ large effect size ( McGough & Faraone, 2009). If a participant omitted a section of the questionnaire, their data for that section was not used for the data analysis.

**Table 1.** Topics Measured

1. The Application Process to Medicine 2. UKCAT and BMAT (United Kingdom Clinical Aptitude Test and BioMedical Aptitude Test) 3. Personal Statement 4. Interviews for Medicine 5. Work Experience and Medicine 6. Volunteer Work and Medicine 7. Life as a Medical Student 8. Life at University 9. Work-Life Balance 8. Ethics in Medicine 10. PBL (Problem-Based Learning) 11. Communication Skills in Medicine

## Results

### School Type Attended by Participants

The majority of participants attended state schools (24, 62%). However, this majority was reduced compared to the proportion of state schools invited (16, 76%). Table B depicts the number of sixth forms invited and the participants in attendance, grouped by school type.

**Table 2.** Number and percentage of sixth forms invited and participants engaged, grouped by school type.

	Number of Sixth Forms Invited (%) *n* = 21	Number of Participants (%) *n* = 39
State Schools	16 (76%)	24 (62%)
Selective Schools	3 (14%)	12 (31%)
Fee-paying Schools	2 (9.5%)	3 (7.6%)
*n* = Total number of sixth forms or participants in attendance.		

### Change in Participant Understanding

Comparison between before and after the intervention showed a statistically significant increase in the levels of participant understanding in all topics covered. All *p*-values of the one-tailed paired t-test were <0.001 and all effect sizes were large (Cohen’s *d* > 0.8). The most notable increase in understanding was in PBL (Increase, 6.69; 95% CI, 5.56-7.81; *p* < 0.001; Cohen’s *d* = 2.89). This was also the topic in which participants understood the least before the intervention (2.60). The smallest increases were in ‘Work Experience and Medicine’ (Increase, 1.69; CI, 0.98-2.41; *p* < 0.001; Cohen’s *d* = 1.07) and ‘Volunteer Work and Medicine’ (Increase, 1.65; CI, 1.01-2.29; *p* < 0.001; Cohen’s *d* = 1.05). These were also the topics in which participants understood the most before the intervention (7.44 and 7.59, respectively).

**Table 3.** Increase in mean participant understanding from pre-intervention to post-intervention questionnaires, using the one-tailed paired t-test and Cohen’s *d* effect size.

Topic	*n*	Pre- Intervention		Post- Intervention		Increase in Mean(95% CI)	*p*-value	Effect Size: Cohen’s *d*
		Mean	SD	Mean	SD			
Application Process to Medicine	37	6.24	2.42	9.00	1.00	2.76 (2.07-3.44)	<0.001	1.49
UKCAT and BMAT	37	5.97	2.95	9.14	0.92	3.16 (2.25-4.08)	<0.001	1.45
Personal Statement	37	7.05	1.81	9.11	1.07	2.05 (1.44-2.67)	<0.001	1.38
Interviews for Medicine	37	5.44	2.13	7.94	1.78	2.51 (1.89-3.14)	<0.001	1.28
Work Experience and Medicine	36	7.44	2.00	9.14	1.02	1.69 (0.98-2.41)	<0.001	1.07
Volunteer Work and Medicine	37	7.59	2.03	9.24	0.89	1.65 (1.01-2.29)	<0.001	1.05
Life as a Medical Student	37	5.14	2.06	8.59	1.44	3.46 (2.83-4.09)	<0.001	1.95
Life at University	36	5.61	2.06	8.47	1.54	2.86 (2.02-3.70)	<0.001	1.57
Work-life Balance	37	5.24	2.19	8.92	1.16	3.68 (2.93-4.42)	<0.001	2.10
Ethics in Medicine	37	6.11	2.02	8.92	1.09	2.81 (2.21-3.41)	<0.001	1.73
PBL	35	2.60	3.13	9.29	0.99	6.69 (5.56-7.81)	<0.001	2.89
Communication Skills in Medicine	37	6.19	2.04	9.00	0.94	2.81 (2.14-3.48)	<0.001	1.59

*n* = Number of entries where both pre-intervention and post-intervention data was available.

95% CI = 95% Confidence Interval, SD = Standard Deviation, PBL = Problem-Based Learning, UKCAT = United Kingdom Clinical Aptitude Test, BMAT = BioMedical Admissions Test.

*p*-value = Result of the one-tailed paired t-test: likelihood that increase in mean understanding was due to chance.

Cohen’s *d* = Increase in mean divided by pooled standard deviation, 0.8 ≤ large effect size

Data is displayed to 2 decimal places as appropriate.

## Discussion

### Key Findings

The intervention was shown to significantly improve ( *p* < 0.001, Cohen’s *d* > 0.8) participant understanding in important areas of the medical school application process. The largest improvements were demonstrated in PBL, Work-Life Balance, Life as a Medical Student, and UKCAT and BMAT. This improved understanding may help counter some of the barriers identified for WP to medicine, such as a lack of support within school environments ( Mathers & Parry, 2009). The authors believe that this model of WP has been shown to provide high-quality support to a large group efficiently, and has potential for replication by other groups. This intervention had several key features:

• No application process for participants.
• Free admission.
• Open to all sixth forms in a high-priority WP area.
• Low-cost to implement.

The study also found that there was a larger proportion of participants attending selective schools (12, 31%) compared to the proportion of selective schools invited (3, 14%). This suggests that initiatives which are open to all sixth forms are more actively taken up by selective school pupils, the inappropriate audience for the intervention. If capacity for an event is limited, it is reasonable to reserve attendance for participants who match WP criteria. As this intervention did not reach capacity, it would have been unreasonable to exclude individuals from selective/fee-paying schools.

However, this intervention was successful in engaging a “cold spot” for medical WP activities, as identified by the Medical Schools Council ( MSC, 2016). Collaboration between medical students and appropriate hospitals therefore appears an effective approach to engaging with identified “cold spots”.

It is important to further understand the efficacy of WP interventions ( HEE, 2014). However, reporting of medical student-led WP initiatives is sparse. To the authors’ knowledge, this was the first study that quantitatively assessed the impact of medical student-led WP initiatives on participant understanding in areas relevant to the medical school application process.

Pre- and post-intervention questionnaires were efficient and effective in establishing the impact of a WP initiative. This approach could be appropriate for student-led initiatives, which may not have the time or resources to devote to more extensive approaches. If deemed successful, this process could be replicated for all post-16 activities. This would enable comparison of such activities and help establish what is effective at widening participation to medicine.

### Manchester Outreach Medics

Since November 2016, the time of the workshop in this study, MOMs has continued to expand and develop. In July 2017, MOMs delivered a large conference for the same target audience, at which attendees delivered presentations of their own. This conference may be discussed in a separate article. In the academic year of 2017/18, BR entered his final year of medical school and handed over the project to Charlotte Auty: a medical student currently intercalating between third and fourth year. In this year, the workshops have reached such a popularity that has allowed WP criteria to be implemented for attendance. The workshops have a similar itinerary, however with more focus on medical school interviews and less focus on work-life balance as a medical student. The initiative has continued to receive positive feedback from attendees and win numerous awards.

### Study Limitations

The quantitative data gathered for participant understanding on topics was assessed using a scale of 0 to 10. Firstly, this is limited as it only assesses perceptions of understanding, which may not accurately assess true understanding. Secondly, participants may have exaggerated positive effects on their understanding, due to response bias or desire to express gratitude to the volunteers. Thirdly, there was no evidence-based guidance or list stating defined topics of participant understanding for such initiatives to improve. The topics included for assessment were those seen as important by the authors and therefore may have not been comprehensive. The study also failed to assess for any long-term impact on participants and improved admissions to medical school.

As the Eventbrite page was public, it is possible that participants involved in the study were not in the target age group or area. Ethical approval to analyse postcode data for this study was not obtained. It could not be determined whether participants themselves were from deprived backgrounds, but simply that participants were from an area that is recognised as being generally deprived. Furthermore, there was an absence of qualitative analysis of the intervention. The inclusion of an evidence-based approach to qualitative data may have aided the measure of intervention efficacy.

### Implications for future practice

The following recommendations for future similar studies were made:

• Obtain ethical approval to analyse pupil demographics.
• Employ long-term follow up on participants to identify the impact on admissions to medical school.
• Employ qualitative analysis to allow a deeper understanding of the impact on participants.
• Apply pedagogy to enhance teaching content and delivery.

The following recommendations for medical widening participation were made:

• Similar activities should be made available to year 12 pupils in all medical widening participation “cold spots”.
• Student-led initiatives and institutions, such as hospital trusts, should collaborate to facilitate effective intervention delivery.
• Standardised pre- and post-intervention questionnaires should be developed to standardise evaluation and enable better comparison of initiatives with similar aims.

## Conclusion

This study has shown that student-led interventions can be effective in improving understanding in key areas for medical school applicants. This reduces barriers to disadvantaged individuals, and promotes widening participation to medicine. The itinerary from this intervention may also provide a possible blueprint for future post-16 widening participation initiatives. The authors suggest that standardised pre- and post-intervention questionnaire should be used by post-16 initiatives with similar aims, to allow comparison between initiatives.

In this study, the intervention required support from external organisations. Organisations may be more likely to collaborate if it is shown that student-led initiatives can be effective. The authors believe that the positive findings from this study have accomplished this. It is hoped that this study will lead to further support for student-led activities, to make them more broadly available and improve widening participation to medicine.

## Take Home Messages

• Student-led interventions can significantly support medical school applicants.
• The itinerary in this study is effective, low-cost and can be employed by other groups.
• More widening participation activities should target high priority areas, or “cold spots”.
• Student-led groups should collaborate with other organisations, such as hospitals.
• Standardised pre- and post-intervention questionnaires could be used by post-16 activities with similar aims, to allow comparison.

## Notes On Contributors

***Ben Ryan*** is a final year medical student at The University of Manchester Medical School. He founded Manchester Outreach Medics in September 2015 and led the organisation until August 2017. He has won university and national awards for his work.

***Amy Kitchen*** is a final year medical student at The University of Manchester Medical School. She was heavily involved in Manchester Outreach Medics, and often co-ran events. She was the lead for the session ‘Simulated Primary Care Centre’, an innovative session used by the organisation at workshops.

***Amy Chan*** is a final year medical student at The University of Manchester Medical School. She volunteered at every event during her time in Manchester Outreach Medics, and played a key role in the branding of the organisation.

***Holly Gibson*** is a final year medical student at The University of Manchester Medical School. She was a regular volunteer at Manchester Outreach Medics events. To further the organisation’s position to inform participants, she independently researched and created a concise, yet thorough, summary of every UK medical school.

***Dr Enam Haque*** is a GP Clinical Lecturer and Academic Lead for Widening Participation at The University of Manchester Medical School. He is involved in many widening participation initiatives, and was extremely important in supporting Manchester Outreach Medics. He also set up the National Medical Schools Widening Participation Forum.

## Declarations

BR has been assessed as part of his medical school curriculum on work pertaining to a different Manchester Outreach Medics event. EH is the widening participation lead within the Division of Medical Education at The University of Manchester Medical School. EH is Chair of the National Medical Schools Widening Participation Forum.

## Appendices

### Appendix Item 1

**Appendix Item 1.** Â– Itinerary

Time	Groups 1-2	Groups 3-4	Groups 5-6
**9:00 - 9:30**	**Registration, Practical Skills and Q&A Stalls** *Participants registered and completed a pre-intervention questionnaire. Prior to the first presentation, participants took part in practical skills, Q&A with medical students and an interactive slideshow presentation.*		
**9:30 - 10:30**	**Welcome Presentations** *The leader welcomed everybody and explained the itinerary for the day. Following that was a 45 minute presentation on ‘Applying to Medicine’ and ‘Life at Medical School’.*		
**10:30 - 11:15**	**Mock PBL (Problem Based Learning) case** *Participants were split up into small groups ran by medical students. Medical students explained what PBL was and participants took part in their own PBL session.*		
**11:15 - 12:00**	**Introduction to Communication Skills** *Staying in small groups, medical students taught participants basic communication skills for interviewing patients, and facilitated practise of these skills.*		
**12:00 - 12:40**	**Presentation on Top Tips for Application** *A 40 minute presentation on ‘Top Tips for Applying to Medicine’.*		
**12:40 - 13:50**	**Lunch**		
**14:00 - 15:00**	**Simulated Primary Care Centre** *Interactive session in which medical students acted as simulated patients and were interviewed by participants.Interviews were followed by teaching surrounding the given scenarios.*	**Personal Statement and UKCAT** *Two half hour interactive presentations on Personal Statement, and UKCAT and BMAT.*	**Work-Life Balance and Medical Ethics** *A presentation from a team of medical students on their experiences at medical school and the importance of work-life balance. This is followed up by group discussions on medical ethics scenarios.*
**15:00 - 16:00**	**Work-Life Balance and Medical Ethics**	**Simulated Primary Care Centre**	**Personal Statement and UKCAT**
**16:10 - 17:10**	**Personal Statement and UKCAT**	**Work-Life Balance and Medical Ethics**	**Simulated Primary Care Centre**
**17:10 - 17:30**	**Wrap-up ** Post-intervention questionnaires completed by participants.		
