# Supplementary material for: Widening participation to medicine: a student-led workshop for medical school applicants
Source: MedEdPublish (2016). 2018 Jun 13;7:130. [Version 1] doi: 10.15694/mep.2018.0000130.1 (PMC10699362; doi:10.15694/mep.2018.0000130.1)
Supplement: Peer review history

# Reviewer report: Reviewer response for version 1

**DOI:** 10.21956/mep.19593.r29091

**Published:** 2019-02-23

**Authors:** Trevor Gibbs

**Affiliations:** 1 AMEE

This review has been migrated. The reviewer awarded 4 stars out of 5 There is still some stigma attached to who should become medical students and doctors of the future- widening participation projects are essential to break down some perceived biases. I enjoyed reading this paper by mainly medical students and it clearly shows how students can be empowered to enhance these widening participation projects. I would prefer that the students looked at longer term evaluations rather than pre and post intervention questionnaires, but despite that I would suggest that this is an important read for all involved in student selection.

Reviewer Expertise:

NA

No decision status is available

# Reviewer report: Reviewer response for version 1

**DOI:** 10.21956/mep.19593.r29092

**Published:** 2018-06-22

**Authors:** Sateesh Babu Arja

**Affiliations:** 1 Avalon University School of Medicine

This review has been migrated. The reviewer awarded 4 stars out of 5 I thoroughly enjoyed reading this paper. I appreciate the efforts of medical schools and medical students to help the students coming from underserved communities and improve their understanding of medical school admission process. This can help with the better diversity of students in the medical program which can further enhance the understanding of students of different cultures and social backgrounds. Authors clearly explained the research methods used and results. They detailed the bias involved and limitations. The main limitation is that framework that was used to assess the understanding of students rather than actually how much they can use or practice from what they have learned. This article is useful for admission committees and administrators of universities.

Reviewer Expertise:

NA

No decision status is available

# Reviewer report: Reviewer response for version 1

**DOI:** 10.21956/mep.19593.r29090

**Published:** 2018-06-17

**Authors:** P Ravi Shankar

**Affiliations:** 1 American International Medical University

This review has been migrated. The reviewer awarded 5 stars out of 5 I enjoyed reading this paper written primarily by medical students. I was not previously aware about widening participation initiatives in the United Kingdom though I had read about similar initiatives in Australia. This is a well written paper and the methods have been adequately described. The itinerary for a session shown in the Appendix is interesting. The session was held in November 2016 but the authors have provided information on events conducted since then. I will be interested in knowing the impact of the Widening participation program on medical school admissions from deprived communities. I assume that the Manchester Outreach Medics (MOMs) continue to provide support to deprived applicants even after their admission to medical school. Similar programs could be replicated in other countries. The manuscript would be of interest to all medical educators and medical student organizations.

Reviewer Expertise:

NA

No decision status is available
